# A Cloud-based Middleware for Self-Adaptive IoT-Collaboration Services

**DOI:** 10.3390/s19204559

**Published:** 2019-10-20

**Authors:** Soojin Park, Sungyong Park

**Affiliations:** 1 Graduate School of Management of Technology, Sogang University, 35 Baekbeom-ro, Mapo-gu, Seoul 04107, Korea; psjdream@sogang.ac.kr; 2 Department of Computer Science and Engineering, Sogang University, 35 Baekbeom-ro, Mapo-gu, Seoul 04107, Korea

**Keywords:** internet of things, self-adaptation, middleware platform, cloud service

## Abstract

The middleware framework for IoT collaboration services should provide efficient solutions to context awareness and uncertainty issues among multiple collaboration domains. However, existing middleware frameworks are mostly limited to a single system, and developing self-adaptive IoT collaboration services using existing frameworks requires developers to take considerable time and effort. Furthermore, the developed IoT collaboration services are often dependent on a particular domain, which cannot easily be referenced in other domains. This paper proposes a cloud-based middleware framework that provides a set of cloud services for self-adaptive IoT collaboration services. The proposed middleware framework is generic in the sense that it clearly separates domain-dependent components from the layers that leverage existing middleware frameworks. In addition, the proposed framework allows developers to upload domain-dependent components onto the cloud, search for registered components, and launch Virtual Machine (VM) running a new MAPE cycle via a convenient web-based interface. The feasibility of the proposed framework has been shown with a simulation of an IoT collaboration service that traces a criminal suspect. The performance evaluation shows that the proposed middleware framework runs with an overhead of only 6% compared to pure Java-based middleware and is scalable as the number of VMs increases up to 16.

## 1. Introduction

In line with rapid development of the Internet of Things (IoT) technologies, many intelligent IoT services for various domains, such as Intelligent Traffic System (ITS) [1], smart home [2], smart building [3], smart city [4,5,6], smart healthcare [7,8] and smart farm [9] are being introduced. Through collaborations among these intelligent systems, new IoT collaboration services that have not been provided in the past, are increasingly becoming a reality. For example, a smart home service detects health problems using various sensors and automatically sends them to the hospital system to help the residents stay healthy in addition to the automatic control of home appliances. Furthermore, it is also possible to analyze the food in the refrigerator and automatically recommend grocery purchases, which in turn connects the refrigerators with the inventory system to maintain appropriate product stocks in the marts. 

As more complex software systems and diverse devices are involved, developing IoT collaboration services requires more time and effort to handle device-specific or domain-specific components, and self-adaptive features such as context awareness and uncertainty. For example, multiple intelligent systems should detect situational context changes from the unintentional behaviors of users and provide appropriate services with no separate explicit operation, which allows us to achieve more by doing less [10]. Besides the context-awareness property, the uncertainty management issue of situational context change must be resolved as well. That is, a collaboration service model among multiple systems in an open IoT environment cannot determine the type and the number of systems to participate in a specific collaboration at design time. It is also difficult to predict when each situational change happens and how much computing power each IoT collaboration service requires. 

Over the past decades, various studies including MAPE-K cycle [7,11,12,13,14], MUSIC [15], DiVA [16], MADAM [17], and Rainbow framework [18] have proposed generic middleware frameworks in various levels. These frameworks allow us to develop software components that monitor (M) context changes, analyze (A) them, plan (P) an appropriate response strategy when a specific issue has occurred, and execute (E) configuration changes at runtime. We call this a MAPE cycle. However, the scope of self-adaptation in these studies [11,15,16,17,18,19,20,21,22] is limited to a single system. Although it is possible to expand the application scope of existing frameworks to develop collaboration services between IoT-based multiple systems, it takes much time and effort to build a self-adaptive IoT collaboration service. For example, developers need to define the role and interface of each software component in great detail. Then, each component should be carefully implemented in order to build a self-adaptive IoT collaboration service. Moreover, other research efforts such as [5,6,8,12,13,14,23,24,25,26,27,28] are artifacts dedicated to specific domains and are difficult to be used as a generic reference model to develop a self-adaptive IoT collaboration service. 

This paper proposes an easy-to-use, cloud-based middleware framework that can be used as a reference framework for self-adaptive IoT collaboration services. This framework clearly separates the MAPE components implementing domain-dependent adaptation logic from the skeleton components for driving the MAPE cycle to support dynamic collaboration reconfiguration. As a result, developers can take advantage of the self-adaptation related capabilities provided by the middleware framework, just by invoking the Application Programming Interfaces (APIs) corresponding to the connector between the general framework and domain-dependent components. Moreover, by creating and managing a dynamically bound Virtual Machine (VM) of the MAPE cycle skeleton and domain-dependent MAPE components on the cloud, it is possible to provide IoT collaboration services anytime and anywhere. In summary, this paper makes the following contributions.

- **Cloudizaion of MAPE cycle for IoT collaboration service**: The proposed middleware is one of the first attempts to cloudize the MAPE cycle for self-adaptive IoT collaboration services. The domain-specific and device-specific components are separated from the implementation as much as possible and the interfaces are provided as cloud service APIs to support the easy development of high-performance self-adaptive IoT collaboration services over the clouds.
- **Development of a convenient web-based interface**: The proposed middleware also provides a convenient web-based interface that allows developers to easily upload their own implemented components, search existing components, register participant systems/devices and launch a VM for starting each IoT collaboration service. These capabilities can reduce the burden of developers and make them focus on enhancing the quality of a particular IoT service component.
- **Implementation and evaluation on a real testbed**: The feasibility of the proposed framework has been demonstrated by simulating an example IoT collaboration service that tracks a criminal suspect. A part of this use case is also prototyped using unmanned patrol cars and CCTVs. It is worthy to note that the proposed framework is not only a conceptual framework but also a workable framework. The benchmarking results show that the proposed cloud-based middleware runs with little overhead of only 6% compared to pure Java-based middleware and is scalable up to 16 VMs compared to standalone applications.

The composition of the paper is as follows. Section 2 reviews related works, while Section 3 introduces a proposed architecture of the cloud-based middleware for self-adaptive IoT-based collaboration. Section 4 presents a use case of an IoT-based collaboration among unmanned patrols and CCTVs belonging to an Intelligent Traffic System (ITS). Section 5 discusses the evaluation results of the proposed middleware framework by running a simple collaboration scenario. Lastly, Section 6 presents the conclusions of the study and future study plans.

## 2. Related Work

Over the past few decades, many self-adaptive middleware frameworks have been introduced. Among them, the most prominent frameworks are TOGAF [29], Rainbow framework, and OSGi (Open System Gateway infrastructure) framework [30]. 

TOGAF [29] is a proven Enterprise Architecture methodology, ensuring consistent standards, methods, and communication. The key to TOGAF is the core model including 8 phases: architecture vision, business architecture, information systems architecture, technology architecture, opportunities and solutions, migration planning, implementation governance and architecture change management. It allows enterprise practitioners to utilize resources more efficiently and assures a design and a procurement specification that can facilitate an open systems implementation. However, it gives a solution for the collaboration of enterprise architectures rather than the collaboration among autonomous multiple systems and devices. The Rainbow framework [18] proposes an external adaptation mechanism that adapts to various system concerns using reusable infrastructure and software architecture. In Rainbow, a conceptual architecture is presented to monitor and detect the need for adaptation in a system. The self-adaptation language describes rule-like constructs (condition-action). When the condition is met, the appropriate action is executed to adapt to the system. It also suggests a language for architecture-based self-adaptation, Stitch [31], which supports the explicit representation of routine human adaptation knowledge using a core set of adaptation concepts. However, the application scope of the Rainbow framework is still limited to a single autonomous system. OSGi [30] is a middleware framework that supports lower-level primitive operations compared to TOGAF or Rainbow. OSGi is a group of standards for writing modular applications on top of the Java Virtual Machine (JVM). After developing an application as a set of bundles, they can be deployed into the runtime system managed by OSGi. OSGi provides a service-oriented development model, but it is often criticized by its complexity, heaviness in the context of a framework, and lack of OSGi tools to manage OSGi bundles [32].

Each of the above self-adaptive middleware frameworks has characteristic contributions, but there is a common limit called solution designed to aim the target of self-adaptation with a single system. Therefore, additional extension work on the adaptation framework by developers is required to support collaboration among multiple systems based on IoT using such frameworks. To solve this problem, recently proposed several studies [7,12,13,14] propose the way to extend the MAPE loop for providing IoT collaboration, which is a more conceptual control loop to organize self-adaptive software using four components: Monitoring, Analysis, Plan, and Execute. [7] proposes a hierarchical computing architecture, HiCH, for cloud and fog enabled IoT-based health monitoring systems, which implements a closed MAPE loop for autonomic system and utilizes machine learning data analytics. [12] analyzes the various architectural patterns for IoT-based crowd monitoring and emergency handling and shows two design patterns chosen are satisfying the expected QoS(Quality of Service) in energy consumption, fault-tolerance, and performance. The proposed self-adaptive IoT architecture has a characteristic in that it could be a bridge and combine the IoT distribution patterns and adaptation logic. [13] presents an architecture with self-healing property for the Internet of Things using ideas from Wireless Sensor Networks applied to a 6LoWPAN network. The study shows the proposed architecture can mitigate four different kinds of attacks of three different layers: machine to machine, network, and cloud. However, the proposed architecture still stays at the conceptual stage, which seems hard to be instantiated into a specific one. [14] proposed four patterns for the composition of autonomic loops, which are leveraging of automata-based modular constructs. They also implemented the proposed framework and present a case study in the field of building automation. However, the proposed framework should be extended for application for IoT services and constructing a specific self-adaptive system by composing the proposed four patterns is up to developers’ effort.

The frameworks [11,15,16,17,18,29,30] are accepted as general solution sets independent of the application domain. While they are applicable to any application domain, they require considerable time for familiarizing with the components in each framework to construct a workable middleware for a specific application domain. In contrast, the studies proposed by [5,6,8,12,13,14,23,24,25,26,27,28] present IoT collaboration examples developed for various domains such as manufacturing [23,27], healthcare [7,8], urban security [24], smart city [4,5,6], smart hospital [25], smart government [26], and cellular communications [28], respectively. 

Among the commented previous studies above, we select ones that suggested an architecture containing specific components for IoT services and compare it according to several distinct characteristics in Table 1. The comparison factors we choose are like the followings:

- Genericity: a generic framework (Generic)/ a specific framework for a target domain (Specific)
- Abstraction level: just a conceptual architecture framework (Conceptual)/ an executable architecture framework for a tangible use case of an IoT collaboration service (Concrete)/ both of Conceptual and Concrete
- Provided as cloud services: The proposed middleware framework is provided as a cloud service (Yes) or not (No)
- Service development in a device-independent environment (Yes) or device-dependent environment (No)

As is shown in Table 1, most of the previously proposed frameworks suggest a generic architecture that defines common layers or components for delivering IoT services. Only [23] presents a concrete middleware framework that could be implemented in a specific development environment but other approaches present conceptual level middleware frameworks. No previous work, listed in Table 1, provides a middleware framework in the form of cloud services, nor does it provide an IoT service development environment that guarantees device-independency. In this study, we aim to propose a generic middleware framework as a cloud service that can be utilized in any device at any time to develop IoT collaboration services. In securing genericity of the proposed middleware framework, we designed explicitly separate layers for domain-dependent IoT collaboration service components and domain-independent components supporting the MAPE cycle. The proposed framework also includes facilities to create a new IoT collaboration service in an easier way by providing a device-independent development environment.

## 3. Cloud-based Middleware Platform for Self-Adaptive IoT Collaboration Services

**Figure 1:**
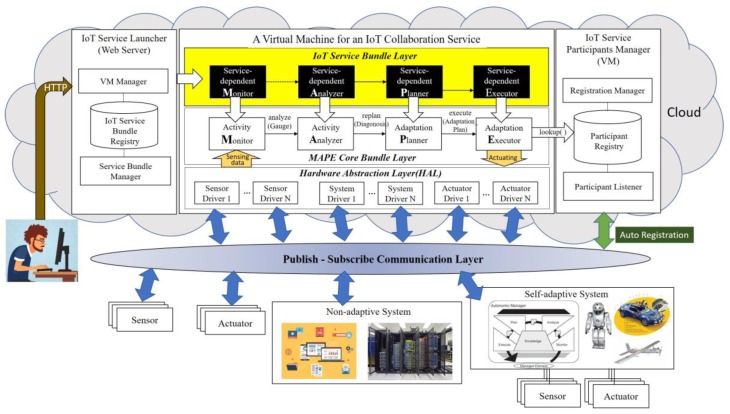
Conceptual architecture overview for cloud-based self-adaptive middleware platform for IoT collaboration services.

Figure 1 shows the overview of a conceptual architecture of the proposed cloud-based middleware framework for the self-adaptive IoT based collaboration among multiple systems. Non-adaptive systems which run on PCs, tablets, or mobile phones and self-adaptive systems such as a robot or unmanned aerial vehicle (UAV) can collaborate to achieve a common service goal. Besides, passive sensors or actuator devices with no separate control unit can also participate in the collaboration as well. The passive actuator may be embedded in and controlled by a single system, or directly receives the commands of the IoT collaboration controller. This communication between various systems and devices is carried out through the *Publish-Subscribe Communication Layer*. As is named, this layer provides a high-performance scalable communication among a large number of IoT devices based on the publish-subscribe communication model. This layer delivers newly published data to consumers who subscribe from multiple but different subsets of sensing data simultaneously. 

Each MAPE cycle for controlling the collaboration of multiple systems and devices is created into one VM in the cloud area. To construct a generic conceptual architecture, we have designed to separate IoT service domain-dependent components from the core MAPE loop components, which are domain-independent parts. Each VM is divided into two layers. One is the *MAPE Core Bundle Layer*, which implements the connectors between the four MAAPE components, the *Publish-Subscribe Communication Layer*, and the *ParticipantRegistry*. The other layer is the *IoT Service Bundle Layer*, which consists of concrete MAPE components required to control a specific IoT collaboration service. The former layer is provided equally to developers, literally, as a middleware framework, and the latter layer includes the components developed by IoT service developers according to the domain requirements. The components belonging to both layers are in the form of OSGi bundles. 

The *IoT Service Launcher* by itself works as one of the VMs created in the cloud area like other VMs for implementing IoT collaboration services. To help developers easily manage the *IoT Service Bundles*, we provide each operation of the *IoT Service Launcher* in the form of a cloud service. The developers can easily perform a series of tasks until the actual IoT service is launched, including the creation/deletion/start/stop of each IoT collaboration service VM. The addition/removal of a specific service bundle can be done by using the cloud service provided by the *IoT Service Launcher* as well. The *IoT Service Participant Manager* provides an auto-registration mechanism of participant systems or devices collaborating in IoT collaboration. Whenever the devices or systems which can participate in an IoT collaboration are power-on, information of the participant is automatically registered to the *Participant Registry*. The address of the *IoT Service Participant Manager* should be saved in the devices and systems before. The *Adaptation Executor* can look up a specific object among the registered systems and devices in the *Participant Registry* as needed.

**Figure 2:**
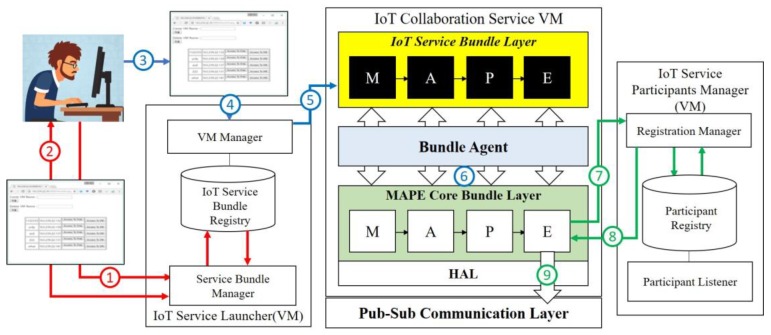
Provided facilities and mechanisms for launching and running an IoT collaboration service.

Figure 2 depicts the web-based facilities and internal mechanism provided by the proposed middleware framework from searching appropriate domain-dependent MAPE components to launching an IoT collaboration service by the components. The following explains each step in more detail.

1. The developer accesses the web page provided by the proposed framework and searches required MAPE bundle components to comprise a designed IoT collaboration service.
2. The search request is delivered to the *Service Bundle Manager*, and the Service Bundle Manager searches the information of bundles stored in the *IoT Service Bundle Registry* and lists them up on the web page.
3. If the developer finds a bundle component that has implemented the function that he/she needs, he/she selects the bundle. If no bundle component of the required function is registered in the *Bundle Registry*, the developer can implement the required bundle component and register it on the web page (the registration of a new bundle is carried out through a separate path through the Service Bundle Manager, which is not shown in Figure 2). After acquiring the component required for the MAPE cycle for running, it is verified whether it provides the intended IoT collaboration service. If the verification result finds no problem, the developer accesses the web page and uploads the prepared MAPE components.
4. The MAPE bundle components selected by the developer are delivered to the *VM Manager*.
5. The components are uploaded to the *IoT Service Bundle Layer*.
6. Each component is bound to the MAPE bundle stub of the *MAPE Core Bundle Layer* by the *Bundle Agent* and one VM is created.
7. The VM working in the cloud requests required participants to the *Registration Manager* when the participation of a new system or device is required in response to an environmental change while performing a MAPE cycle
8. The *Registration Manager* searches the *Participant Registry* and provides the information on new systems/devices to participate in the collaboration.
9. Among the MAPE components, the Executor component, which executes practical collaboration re-configuration, drives the new system/device through the *Publish-Subscribe Communication Layer* by using the acquired information.

**Figure 3:**
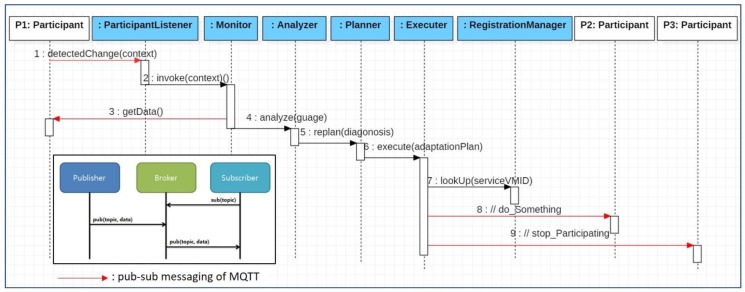
Provided pattern for communication between participants and Virtual Machine (VM) in cloud.

The proposed middleware framework also provides developers with protocol templates utilizing the publish-subscribe model between participant systems/devices, between participants and VMs in the cloud, or between VMs, thus minimizing the development overhead as well. Figure 3 below shows a sequence diagram for a pattern in which participating systems in collaboration communicate through the proposed middleware framework while an IoT collaboration service is provided. 

The specified communication is like the following: a MAPE cycle is activated by an environmental change detected by a particular participant (P1). As a result of analyzing the detected change, a new reconfiguration plan is executed, which instructs a participant system(P2) to perform a specific operation (// 8. Do something) and makes a new system(P3) participate in the collaboration. Among the objects at the top of the sequence diagram, shaded objects indicate the bundle components that are included in the VM and operate in the cloud, whereas the non-shaded objects indicate the participant systems or devices in collaboration through the *Publish-Subscribe Communication Layer*. The red-colored messages denote messages that are sent or received through the *Publish-Subscribe Communication Layer*. In the pub-sub messaging protocol marked at the bottom left of the sequence diagram, the publisher is mapped to a caller object that calls the message and the subscriber is mapped to a callee object to whom a message is called. Every practical operation requires repeated communication initialization steps such as sub(topic)→pub(topic, data)→pub(topic, data) according to the pub-sub messaging pattern. The purpose of wrapping these repeated messaging sequence with an operation is to reduce the development load of the developer by hiding the information which could be patterned.

The proposed middleware framework is a generic framework which can be adopted by any kinds of IoT collaboration domain and it has the following three unique features:

1. Registration and search of MAPE bundle using a convenient web interface are supported.
2. Minimize the development efforts by separating concerns from the implementation: explicit design of layers for the domain-independent parts and device-independent parts in the architecture makes developers focus on creating only a specific IoT collaboration specific components.
3. Auto-registration of candidate systems and devices is also supported.

## 4. A Use Case for Arrest of a Criminal Suspect by Collaboration of Unmanned Patrol Cars and CCTVs in an IoT Network

The IoT-related solutions introduced through literature until now belong to the public service domain related to personal safety such as smart city, smart transportation, and smart healthcare. In this vein, the present study also selected the smart public security service as the target domain of the proposed middleware framework as well. Among the smart public security service cases, we design a criminal suspect arrest use case by a collaboration of multiple unmanned patrol cars and CCTVs. The collaboration designed in the use case is an IoT-based self-adaptive service, in that the scope of systems and devices to participate in the criminal suspect arrest is variable depending on the severity of the crime and the escape route of the criminal suspect cannot be predicted beforehand.

**Figure 4:**
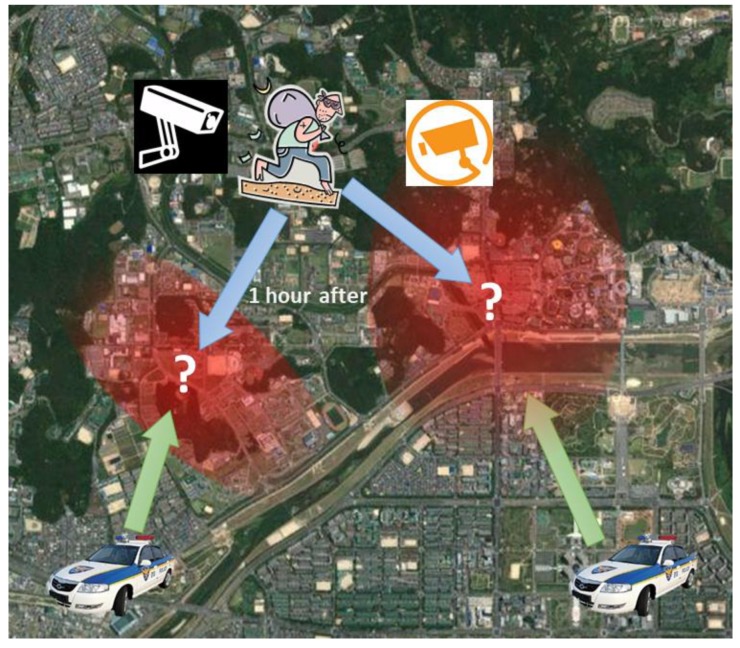
A use case for arrest of a criminal suspect by the collaboration of unmanned patrol cars and CCTVs.

The following is a scenario specification example for the criminal suspect arrest use case depicted in Figure 4.

1. A theft case was registered in the police agency system, and according to the reporter, the theft suspect is not riding a car.
2. The police agency system registers the theft case report point as the initial location of the theft suspect in global knowledge and creates a collaboration (VM) for tracking the theft suspect. A log for theft suspect tracking collaboration of a similar case in the global knowledge is searched and registered in the global knowledge of the newly created collaboration.
3. The current location tracking radius of the theft suspect is calculated based on the difference between the theft case occurrence time and report time. Then the impression and tracking radius of the theft suspect is sent to the ITS (Intelligent Transportation System). All the images of CCTVs in the tracking radius are analyzed and the transmission of photographs of people who match the impression of the theft suspect is requested.
4. The police agency system notifies the place of theft case occurrence to unmanned patrol cars within +10 km of tracking radius and the locations of traceable cars are identified.
5. The unmanned patrol cars participating in the tracking activate *Participant Listener* to update the current location of the theft suspect. Whenever the *Participant Listener* detects update, the tracking route is reset by the shortest distance between the escape route of the theft suspect and the current location of oneself through analysis of similar tracking log data in global knowledge.
6. The ITS receives real-time images from CCTVs within the tracking radius, compares them with the impression of the theft suspect, and identifies the current location of the theft suspect. If it is determined that the theft suspect has been captured by a CCTV, the current location of the theft suspect is updated.
7. The CCTV activates the *Participant Listener* for the update of the current location of the theft suspect. If the current location of the theft suspect is updated, the CCTV determines whether the current location of the theft suspect is within the visible range of itself. If it is, the camera direction is adjusted toward the current location of the theft suspect and sends the images of the suspect to the ITS until the suspect movers out of its visible range.
8. Steps 4 to 7 are repeated.
9. If the theft suspect is besieged by unmanned patrol cars, or if an official of the police agency decides to close this case because further tracking is no longer meaningful, the use case is terminated.

**Figure 5:**
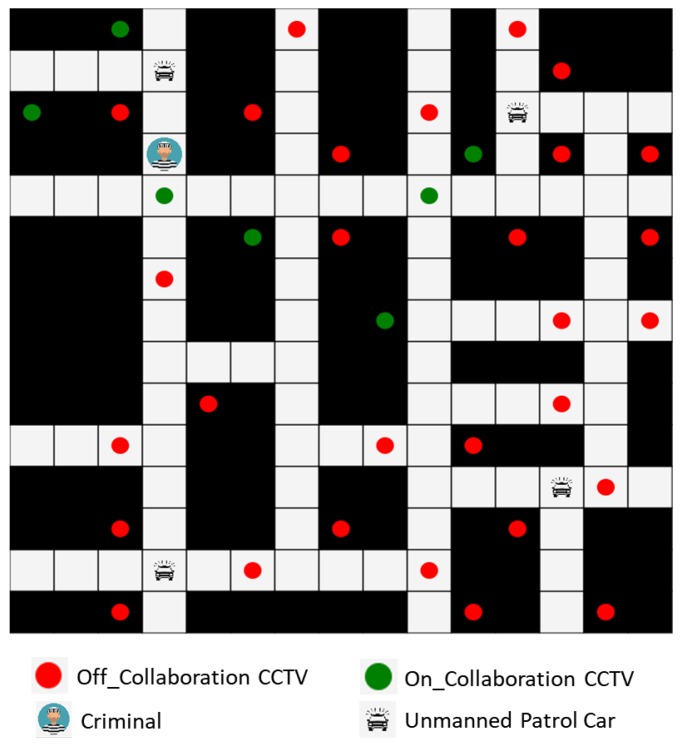
A Simulated application for the arrest of a criminal suspect by a collaboration of unmanned patrol cars and CCTVs.

For the selected scenario to be carried out successfully, not only the QoS (Quality of Service) issue of the middleware framework proposed in this paper but also the functionality of the MAPE component itself that directly affects goal achievement through actual collaboration must be satisfied. However, it is difficult to test the goal achievement of the scenario by activating multiple unmanned patrol cars and CCTVs in the streets. Therefore, to verify the reliability for the achievement of the collaboration goal of the MAPE component itself, we have constructed a simulation environment of criminal suspect arrest in Java, and the GUI screen for the simulated environment is shown in Figure 5. A certain size of the map can be defined on the GUI screen, and the map is fixed during a collaboration for the same criminal tracking. The current location of the criminal and the locations of the unmanned patrol cars participating in the collaboration are indicated on the map, and the locations of CCTVs on the streets identified by the ITS are marked as circles on the map. Green CCTVs means that they are now sending images for criminal tracking, that is, participating in the IoT collaboration. The red CCTVs mean that they are monitoring traffic as their original mission and not participating in the collaboration. The installation locations of the CCTVs means at the time when the map is initialized during the simulation starts, and the number of unmanned patrol cars participating in the collaboration for criminal tracking can be also specified at the same time. The initial location of the criminal and the initial location of each unmanned patrol car are randomly changed whenever the simulation task is performed in the same environment.

**Figure 6:**
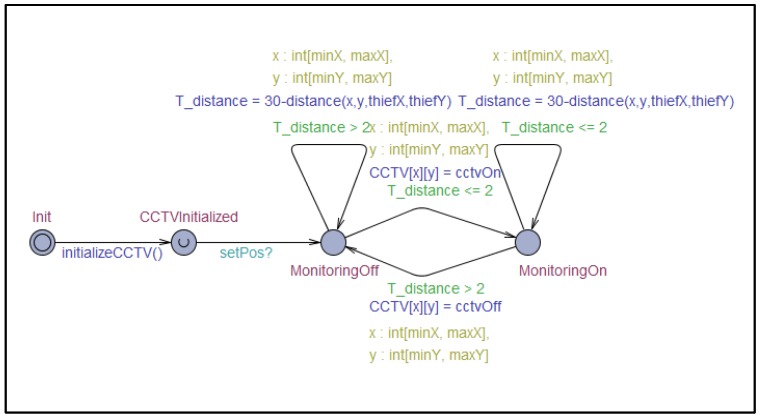
Timed automata for CCTV participating in IoT collaboration for the arrest of a criminal suspect.

**Figure 7:**
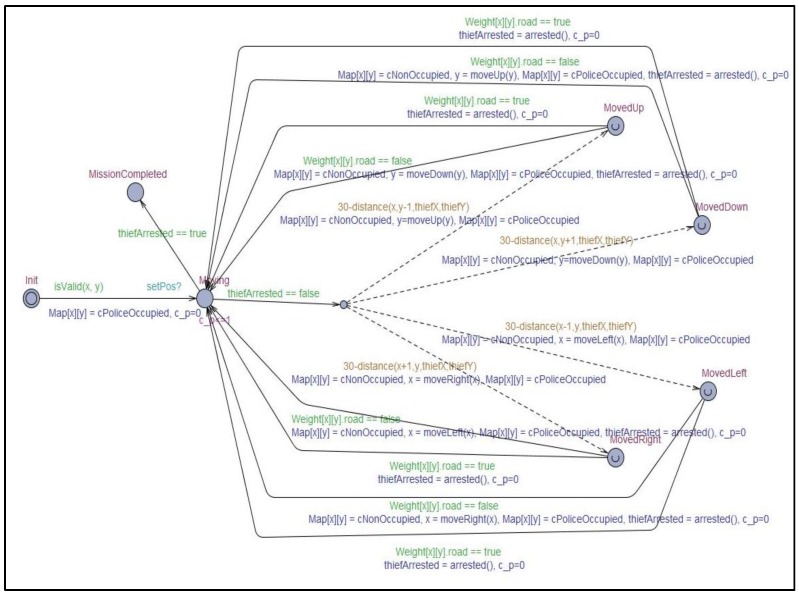
Timed automata for unmanned patrol car participating in IoT collaboration for the arrest of a criminal suspect.

To verify within which degree of reliability the collaboration goal of criminal suspect arrest can be achieved by the collaboration model between unmanned patrol cars and CCTVs included in the simulation, the behavior logic included in the simulation model implemented in Java was expressed as a model in the form of timed automata using UPPAAL [33] as shown in Figure 6 and Figure 7. UPPAAL is an integrated tool environment for modeling, validation, and verification of real-time systems modeled as networks of timed automata. As shown in Figure 6 and Figure 7, the UPPAAL can be used to design and simulate the MAPE cycle embedded in the single systems participating in the collaboration and message passing between models representing single systems. Furthermore, it is also possible to derive the result of verification for specific queries in the designed timed automata. 

To verify the degree of achievement of the IoT collaboration goal for criminal suspect arrest, simulations of the designed model were performed 1000 times while increasing the number of unmanned patrol cars on a 15 × 15 sized map from 1 to 10. Table 2 lists the changing trend of criminal arrest probability. As the number of unmanned patrol cars participating in collaboration increased from 1 to 4, the arrest rate increased sharply. When the number of unmanned patrol cars increased to 6 or more, the arrest rate was 100%.

Space and cost constraints are too high to prove the feasibility of the proposed middleware framework through the demonstration of IoT service among real unmanned patrol cars, police agency systems, and CCTVs controlled by the ITS. Therefore, we evaluated the functionality of the MAPE components implementing the IoT collaboration service through the construction of a simulated application and a timed automata model considering various environmental variables. The results in Table 2 demonstrate that the MAPE components implemented for the IoT collaboration in a cloud satisfy the functionality of the service of criminal suspect arrest, although the scenario is simulated in a Java application.

Furthermore, we performed prototyping of limited scope to verify the effectiveness of the supportability of the middleware framework for actual communication between devices. We constructed a testbed for the IoT based collaboration by developing prototypes of the unmanned patrol car and the CCTV, which are specified in the arrest of a criminal suspect scenario. We developed 2 unmanned patrol cars with a smartphone and an Arduino smart car. We also made 6 CCTVs with WebCams, Arduinos and Rasberry PIs. Table 3 lists the hardware specifications and development environments for each developed component of the prototypes in detail. 

**Figure 8:**
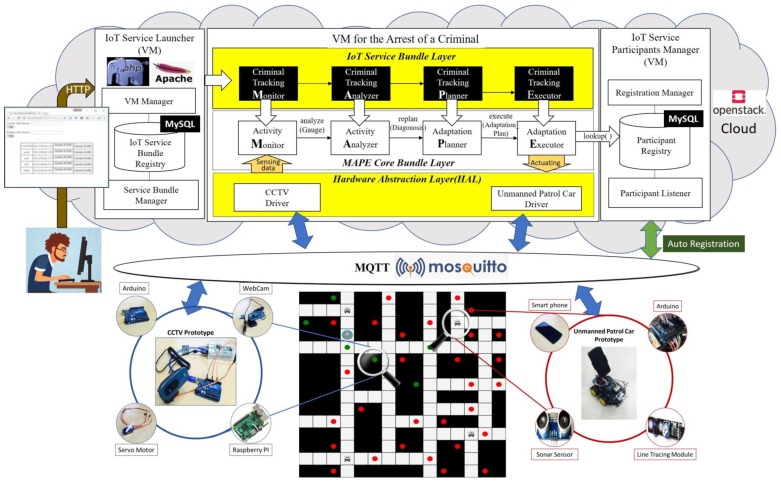
The constructed testbed of the collaboration for implementing the scenario for arresting a criminal suspect.

As is depicted in Figure 8, the proposed reference architecture in Section 3 was instantiated as a concrete one to control the collaboration between 2 unmanned patrol cars and 4 CCTVs. In Figure 8, the highlighted components with black color are instantiated parts for this prototyped testbed. First, we implemented MAPE bundles for criminal suspect tracking and uploaded them to OpenStack [34] cloud via a web page. OpenStack is an open-source cloud platform for private/public clouds and the *IoT Service Launcher* is implemented as the form of a cloud service based on PHP [35] and Apache [36]. The uploaded bundles are registered in *IoT Service Bundle Registry*, and then, the bundles are bounded to the *MAPE core bundles*, which will compose a new VM to control the collaboration. IoT Service *Bundle Registry* and *Participant Registry* were constructed in MySQL 8.0 server. We implemented the *Publish-Subscribe Communication Layer* in the reference architecture using the Mosquitto MQTT (Message Queuing Telemetry Transport) Broker [37], which is a lightweight open-source message broker and one of the most notably used protocols in IoT applications [38].

**Figure 9:**
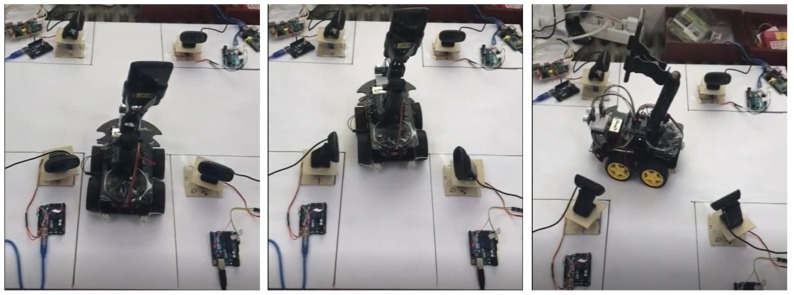
Demonstration of IoT collaboration between an unmanned patrol car and CCTVs.

Among the various potential situations in the simulated application introduced in Section 3, a spot of tracking scenario selected for prototyping is as follows: In a situation where two unmanned patrol cars are chasing a criminal suspect approaching a crossroad, collaboration among devices is performed by involving four CCTVs installed around the crossroad in the collaboration and adjusting the directions of the webcams appropriately to keep track of the escaping criminal suspect continuously. Figure 9 shows the criminal suspect tracking scene at the crossroad of the testbed that was actually constructed. The criminal suspect escaping from the right side to the left side of the crossroad was detected by CCTVs and notified to the other participants. In this situation, the unmanned patrol cars located at the bottom of the crossroad are moving toward the direction of the criminal suspect and the CCTVs on both sides of the bottom of the crossroad are changing the direction of the camcorders as shown in Figure 9. This collaboration among the devices was implemented by using the communication API redefined in the proposed middleware framework based on MQTT.

## 5. Evaluation

In the previous section, we have already shown a tangible scenario for the arrest of a criminal suspect can accomplish the goal by using a simulated application and a probability model checking environment. And, to prove the feasibility of the proposed middleware framework, we have also shown a demonstration of IoT collaboration among multiple participating systems which are supported by the implemented middleware framework.

**Figure 10:**
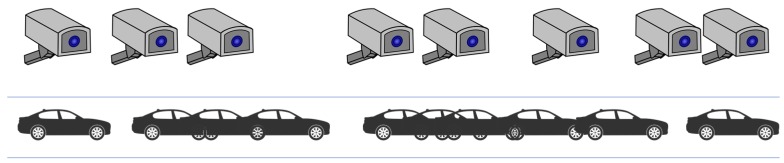
A scenario for the collaboration of CCTVs for tracking a car.

In this section, we discuss the quantitative QoS (Quality of Service) evaluation results. We set the two QoS factors to measure the quality of the proposed middleware framework: overhead and scalability derived from the cloudization of the middleware services. In the scenario discussed in Section 4, it is hard to take apart the performance of the middleware itself from the quality of the whole service as there are a lot of factors (quality of a network, hardware spec., and sensitivity of sensors, etc.) to affect the quality. Therefore, we implemented a much simpler scenario of IoT collaboration, which is a collaboration of sixteen CCTVs to track the movement of a particular car. The collaboration rule is simple. Each CCTV controller monitors the status of neighbors on both sides and turns on their webcams when one of the neighbors is on. After turning on the webcam, CCTV controllers analyze the captured images and continue shooting when the target car is captured in the video. If the target car is not captured in the video for successive 10 s, the controller turns off the webcam and start to monitor neighbors’ status again. Figure 10 depicts the scenario.

### 5.1. Middleware Overhead from Cloudization of the IoT Collaboration Control 

One feature of the middleware framework proposed in this study is that the bundles for performing MAPE cycles to control IoT collaboration are provided as VMs in a cloud. This feature provides the advantage that IoT collaboration services can be activated anytime, anywhere, but it can cause additional communication overhead through the middleware. To analyze those middleware overheads from cloudization of the IoT collaboration control, we implemented the middleware for supporting the collaboration of CCTVs to track a car in the following three different versions.

1. Pure Java application: Implementation as a pure Java application in each CCTV controller (local system)
2. OSGi bundles in a local system: Implementation as an OSGi-based bundle in each CCTV controller
3. OSGi bundles in OpenStack: Implementation of a VM corresponding to each CCTV controller in OpenStack. Each MAPE component is an OSGi-based bundle.

**Figure 11:**
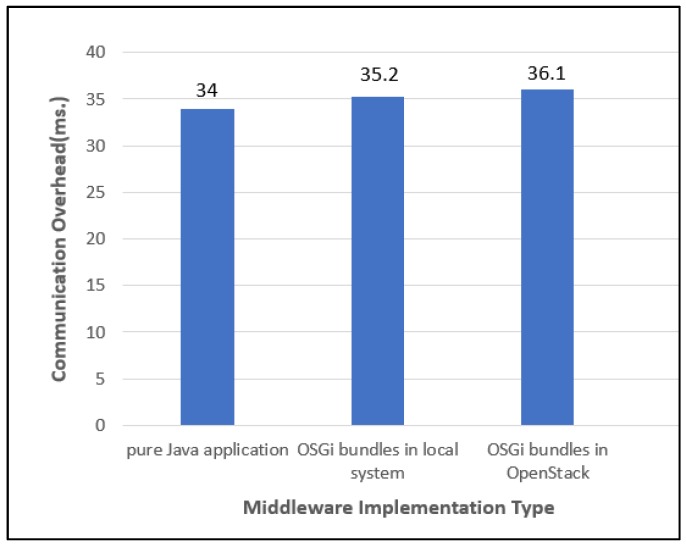
The observed overhead of the middleware according to the implementation types.

The graph in Figure 11 shows the average values of the time consumed for communication between the middleware framework and devices measured during the performance of the CCTV collaboration scenario. As expected, the time increased in the ascending order of pure Java application, OSGi bundles in a local system, and OSGi bundles in OpenStack. However, it can be found that the middleware overhead when the MAPE components are implemented in OSGi bundles and served on OpenStack has increased by only 2.1 ms compared to that of the case to implement the MAPE components with a pure Java class and served on the local system. In other words, only 6% of additional overhead has occurred from the cloudization of the middleware services. It can be interpreted as an acceptable level, considering the ubiquitousness that can be obtained by the cloudization of the MAPE implementing components or the provision of an environment that facilitates the development of IoT collaboration services by providing an API for wrapping major communication sequences.

### 5.2. Scalability

**Figure 12:**
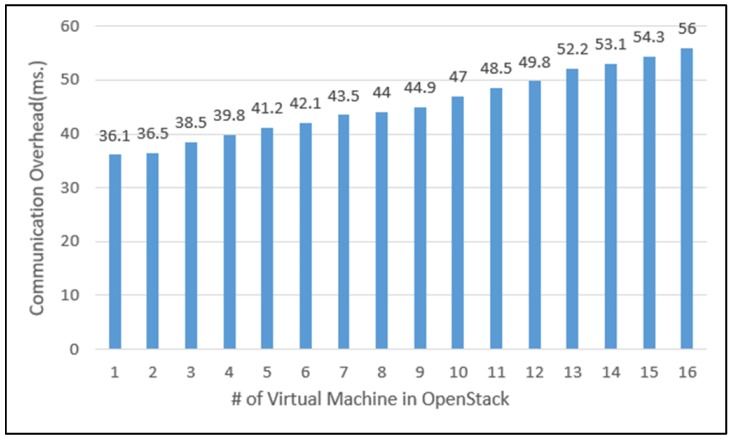
The communication overhead of the middleware according to the increase of VMs in Cloud.

The number of participating systems or devices in open IoT collaboration cannot be restricted in advance. For this reason, scalability is a key QoS that must be satisfied by the middleware framework that supports open IoT collaboration. To estimate the increasing trend of overhead that may occur when multiple domains are performed in a cloud, we measured communication overhead between VMs and CCTVs through the middleware while increasing the number of VMs corresponding to CCTV controllers from 1 to 16 in a collaboration scenario among CCTVs. The average values of the measured communication overhead are shown on the graph in Figure 12. When there was one VM in the OpenStack cloud, the communication overhead was 36.1 ms, and as the VMs increased one by one, the overhead also increased. However, the average overhead when 16 VMs operated was 56 ms, which only increased by 19.9 ms compared to one VM. In other words, a rapid increase in overhead with the increasing number of VMs was not observed. 

**Figure 13:**
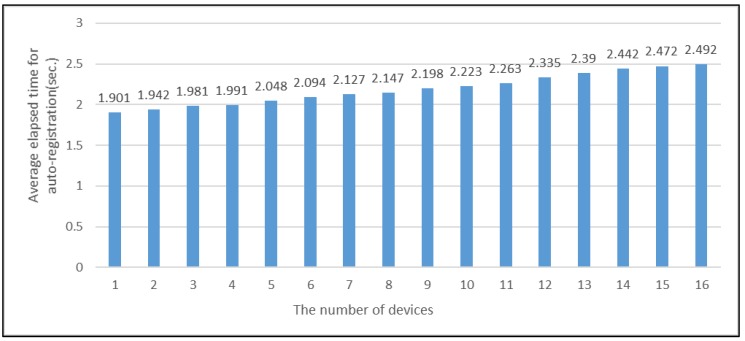
The average elapsed time for auto-registration of devices.

The proposed middleware framework support auto-registration of each device, which is a candidate for a participant of an IoT collaboration service. As the number of devices increased, we verified how much averaged time it would take for the middleware framework to automatically register each device at the moment the device is powered on. As in the previous experiment, we added 16 CCTV devices incrementally and measured the average time for registering each device. The result is depicted in Figure 13. When there existed only one CCTV, the elapsed time from the moment of turn-on of the CCTV to the time when the information of the CCTV is registered as a candidate device was 1.901. Meanwhile, when the number of CCTVs reached 16, the average elapsed time for the auto-registration of each CCTV was 2.492 s, which only increased by 0.591 s. Like the result of the previous experiment, any rapid increase in the average time of auto-registration was not observed. 

Our experiments on the scalability of the proposed middleware framework were limited within 16 CCTVs as there are spatial and budgetary limitations in the construction of a testbed. However, as we found, the result of the two experiments can show that the proposed middleware framework does not have, at least, a serious issue in terms of scalability.

## 6. Conclusions and Future Work

In many cases, IoT collaboration services between various systems and devices are continuously provided even when we do not feel them in our daily lives. As a result, the support of middleware framework is essential to detect environmental changes and to re-configure IoT collaboration services as a response to the detected environmental changes at runtime. Although various frameworks supporting self-adaptation have been introduced, it is difficult for experienced developers to easily apply most of the previously introduced frameworks to IoT-based collaborative services because of the issues such as the scope of self-adaptation, generality of applicable domains, and the level of abstraction of the framework itself.

To solve this problem, this paper proposed a middleware framework as cloud services, which make developers easily utilize the MAPE cycle in any device, at any time, even though they are not familiar with the mechanism for running the MAPE cycle. It was possible due to the separation of domain-independent mechanisms from other implementation of the IoT collaboration services in the proposed framework. Furthermore, the auto-registration provided by the proposed framework allows developers to focus on implementing IoT collaboration services that are developed without caring about the type of participating devices. The feasibility of the proposed cloud-based middleware framework was demonstrated both by the simulation and by prototyping of a case of a collaboration service among unmanned patrol cars and CCTVs to arrest a criminal suspect. Through this feasibility test, we have shown that the proposed media frameworks can be easily instantiated as frameworks for developing IoT collocation services while being useful as a reference architecture framework. The key quality attributes in an open IoT environment such as scalability and communication overhead were evaluated by using a simple case of the collaboration between CCTVs for tracking a moving car. The amount of overhead incurred by cloudization of the middleware framework was not so severe considering the convenience of services provided by the proposed framework. 

As future work, we are planning to upgrade the QoS of middleware frameworks by applying the middleware framework proposed in this paper to various IoT collaboration service domains. It is expected that it will be able to contribute to reducing developers’ development burden when constructing a new IoT collaboration service by identifying and reflecting the domain-specific collaboration pattern in the framework itself.

## Figures and Tables

**Table 1:** Comparison of previous research and the proposed middleware framework for Internet of Things (IoT) services.

Worked by	Genericity of Architecture (Generic/Specific)	Abstraction Level (Conceptual /Concrete)	Provided as Cloud Services	Device-Independent IoT Service Dev. Env.
[4]	Generic	Conceptual	Partially Yes	No
[7]	Generic	Conceptual	No	No
[12]	Generic	Conceptual	No	N/A
[13]	Specific	Conceptual	No	No
[14]	Generic	Conceptual	No	No
[23]	Specific	Concrete	No	No
[24]	Generic	Conceptual	No	No
[25]	Specific	Conceptual	No	No
[26]	Too Generic	Too Conceptual	No	No
Proposed	Generic	Both	Yes	Yes

**Table 2:** Probability of arresting a criminal suspect according to the increase of unmanned patrol cars.

# of Participating Unmanned Patrol Car	Probability of Arrest (%)
1	19.8
2	32.3
3	75.2
4	97.4
5	98.7
6	100
7	100
8	100
9	100
10	100

**Table 3:** Hardware components and development environments for the prototypes.

Prototype	Software Component	Hardware Specification	Development Environment
Unmanned patrol car	Controller	LG Nexus 5X phone	Java/Eclipse
	Sensor	HC-SR04 sonar sensor, Arduino Uno R3	Sketch/Arduino IDE
	Actuator	DC geared motor, Arduino Uno R3	Sketch/Arduino IDE
CCTV	Controller	Raspberry PI B2	C/Ubuntu MATE
	Sensor	Logitech C270 Web Cam, Arduino Uno R3	Sketch/Arduino IDE
	Actuator	Servo motor, Arduino Uno R3	Sketch/Arduino IDE
MAPE components	MAPE Controller	Linux Server	Java/Eclipse
